# Role of the skin microbiome in atopic dermatitis

**DOI:** 10.1186/2045-7022-4-33

**Published:** 2014-10-17

**Authors:** Alexander Salava, Antti Lauerma

**Affiliations:** Department of Dermatology and Allergology, Helsinki University Central Hospital and University of Helsinki, Meilahdentie 2, Helsinki, 00250 Finland

**Keywords:** Atopic dermatitis, Skin microbiome, Cutaneous microbial diversity

## Abstract

Atopic dermatitis (AD) is a common inflammatory skin disorder and characterized by abnormalities in both skin barrier structures and alternations of the immune response. Molecular genetics have dramatically changed our vision of the micro-organisms colonizing the human skin and recently well-documented changes in the skin microbiome in atopic dermatitis have become evident. The microbiome shifts have been primarily documented during disease flares and localized to sites of disease predilection, e.g. folds or facial area. In contrast, active treatment has been associated with a recolonisation and higher cutaneous microbial diversity. Additionally to the known dysfunctions in barrier function of the skin (e.g. filaggrin mutations) and immunologic disturbances (e.g. Th_2_-shift), evidence is rising that atopic dermatitis is also connected to a dysbiosis of the microbial community without an invading pathogen. In the future the investigation of the patient’s skin microbiome may have a foothold in the clinician’s diagnostic repertoire and treatment of atopic dermatitis.

## Introduction

### The skin microbiome

Human skin can be considered as a complex ecosystem. An area of approximately 2.0 square meters can accommodate a great variety of habitats ranging from wet skin in the axillary folds to drier areas of the shins. As primarily an interface between the outside environment the skin is inhabited by a number of micro-organisms such as bacteria, fungi, mites and viruses [1]. The current understanding is that the majority of these micro-organisms is harmless and can even provide protection against harmful effects. The interaction between the host and the skin microbiome can be viewed as a symbiosis. For the protection from pathogenic organisms the commensal microbial flora receives as a compensation an area of skin to colonize and ecological niche in the host [2]. Additionally skin microbes have been found to play an important role in the modulation of the host’s cutaneous native and adaptive immune system. Communication with apathogenic commensal microbes is likely partly training cutaneous T-cells to recognize correct antigens and develop an adequate immune response [3].

### Understanding the skin as an ecosystem

From a simplified perspective, it is primarily the physical and chemical characteristics of a particular skin area that determine the microbial flora. In particular skin thickness, number of skin folds as well as the amount of cutaneous appendages, i.e. sweat and sebaceous glands, hair follicles and their activity have a substantial effect on the skin microbiome. On the other hand, host characteristics like age, gender and moreover immunological factors have an influence on the composition of the microbial flora, so that the normal resident commensal flora has been shown to vary significantly at different body sites [4].

Some differences have been in part explained by the physiological and anatomical differences between men and women's skin, such as sweat and sebum quantity, as well as differences in sex hormone production. Furthermore occupation, environmental factors, such as clothing and the use of topical or systemic antibiotics, cosmetics, soaps, personal care products and moisturizers are also possible factors that affect the skin micro-organisms. Studies of habitat and the influence of cultural aspects of society have shown that high-temperature and moisture (e.g. seen in areas of warm humid climate) is associated with increased amount and a broader spectrum of bacteria on the skin [5]. Interestingly, on dry skin a dominance of gram negative bacteria has been observed [2].

The skin in general is colonized with an abundant number of bacterial species and colonization of micro-organisms takes place immediately after birth [6]. A very interesting area for future research will be the development and eventual establishment of the cutaneous microbial diversity during the first years of life and possible connections to skin diseases like atopic dermatitis [7].

## Interaction of microbiome and host-immune system

### The immunological barrier of the skin

It has become apparent that the skin represents not only a physical barrier but also as an immunological one and that the composition of the microbiome is influenced by the host’s native and adaptive immune system. A critical function of the epithelium is the cutaneous innate immune system, which orchestrates when and how the immune system should respond to commensal or pathogenic microbes. The epidermis directly borders with the environment and expresses e.g. many pattern recognition receptors (PRRs) that make it a key player in cutaneous innate immune responses to skin infections and injury. Thus, the cutaneous innate immune system can be viewed as a key determinant of the immunologic barrier functions of the epidermis. Malfunctions in this system can lead to an inadequate host response to a pathogen or a persistent inflammatory state [8].

Secondly, the immune cells of the adaptive immune system, especially intraepithelial lymphocytes, have showed to be involved in maintaining the integrity of the epithelial surfaces. They seem to contribute towards the tolerance to commensal apathogenic micro-organisms and to adequate immune responses against harmful organisms and their products. Complex and prolonged interactions between the skin microbiome and host immune system of skin and mucous membranes have been described. Recent publications have gained further insight to the role of the cutaneous adaptive immune system by examining the microbiome of patients with rare primary immunodeficiencies, e.g. Hyper-IgE-syndrome or Wiskott-Aldrich-syndrome [9]. Interestingly, while there is a great difference in the specific immunologic defect, a shared hallmark is a clinical picture imitating atopic dermatitis.

### Effect of the microbiome on the host’s immune system

Interactions with skin commensals are now understood as a key in developing cutaneous immunity. In atopic dermatitis some data exist that the cutaneous microbes can vice versa, have an impact on the hosts immune system. One possibility is that the skin microbes may alter the activation of cutaneous immunity. The elevated serum IgE-levels in these patients may be related to allergy or atopy and may also reflect an aberrant response to a colonizing microbe [3].

## New molecular approaches to examine cutaneous microbial flora

New innovations in sequencing technology and molecular diagnostic methods, Next Generation Technologies, have revolutionized our understanding of the skin microbiome in recent years and have enabled explorations of the complexity of the immense human-associated microbiota in recent years. They have also emphasized the balanced interaction between the microbes and the host [10].

As a result of genomic studies based on 16S ribosomal RNA metagenomic sequencing it has become evident that the skin colonization of microorganisms has a greater diversity and variability than previously assumed [11, 12]. Based on 16 S ribosomal RNA polymorphisms the skin bacteria can be divided into four phyla: *Actinobacteria, Firmicutes, Proteobacteria and Bacteroidetes*. These seem to prevail also on the adjacent mucous membranes. There have also been reports of detection of 16S ribosomal RNA in deep skin layers, indicating that the microbiome could extend to subepidermal compartments of normal skin [13]. The proportions of these different bacterial phyla seem to vary greatly in different areas of the skin. Current knowledge indicates that the proportions and the established microbiome, however, remain surprisingly stable during the life span and in an individual [14].

However, the used methods of molecular genetics have some disadvantages. For example they are not able to distinguish between bacterial 16 S rRNA genes, which are derived from living or dead organisms. In conclusion they represent in vitro tests. As the skin is exposed to a multitude of environmental influences and micro-organisms, it is impossible to determine which of the identified species represent transient and permanent colonization. Additionally it is not possible to acquire information about sensitivity to antibiotics sensitivity, e.g. to separate methicillin-sensitive and methicillin-resistant *Staphylococcus aureus* (MRSA) species [15].

## The microbiome in atopic dermatitis

### The complex pathogenesis of atopic dermatitis

**Figure 1:**
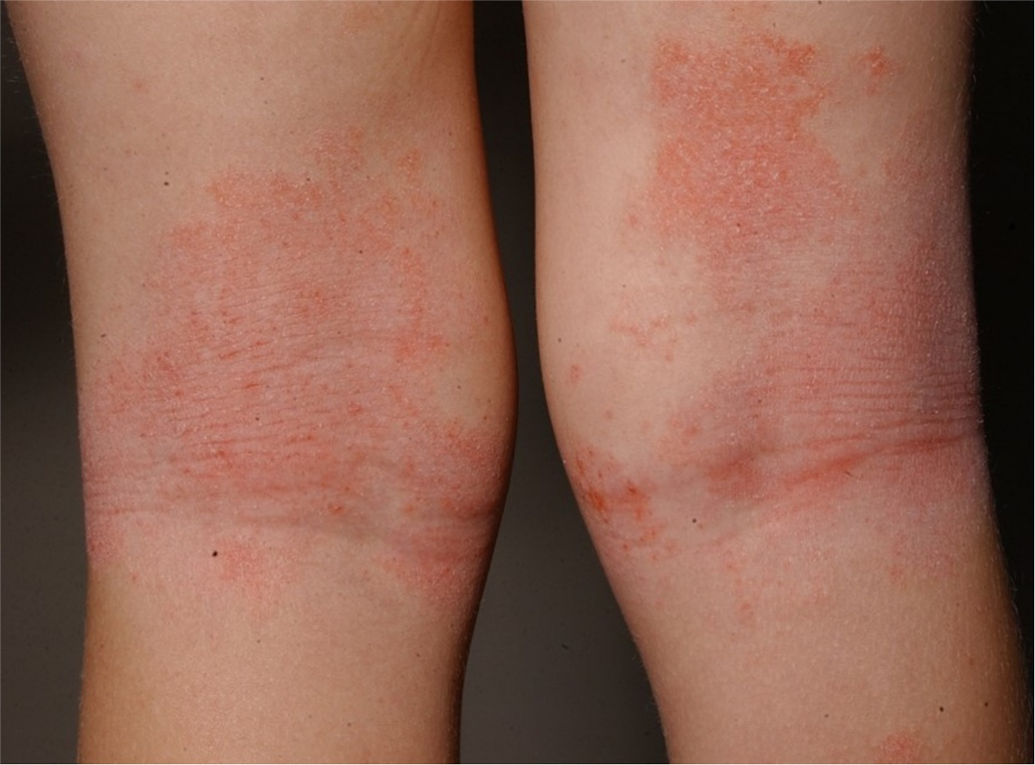
**Typical lichenified atopic dermatitis in the kneefolds.**

Atopic dermatitis is the most common inflammatory skin disorder and characterized by abnormalities in both skin barrier structures (e.g. filaggrin gene loss-of function mutations), a robust Th_2_-response to environmental antigens, possible deficiencies in innate immunity and a well-documented alternation of the skin microbiome [16]. Many of these abnormalities may occur as the consequence of epidermal dysfunction (Figure 1).

### Changes in the skin microbiome

**Figure 2:**
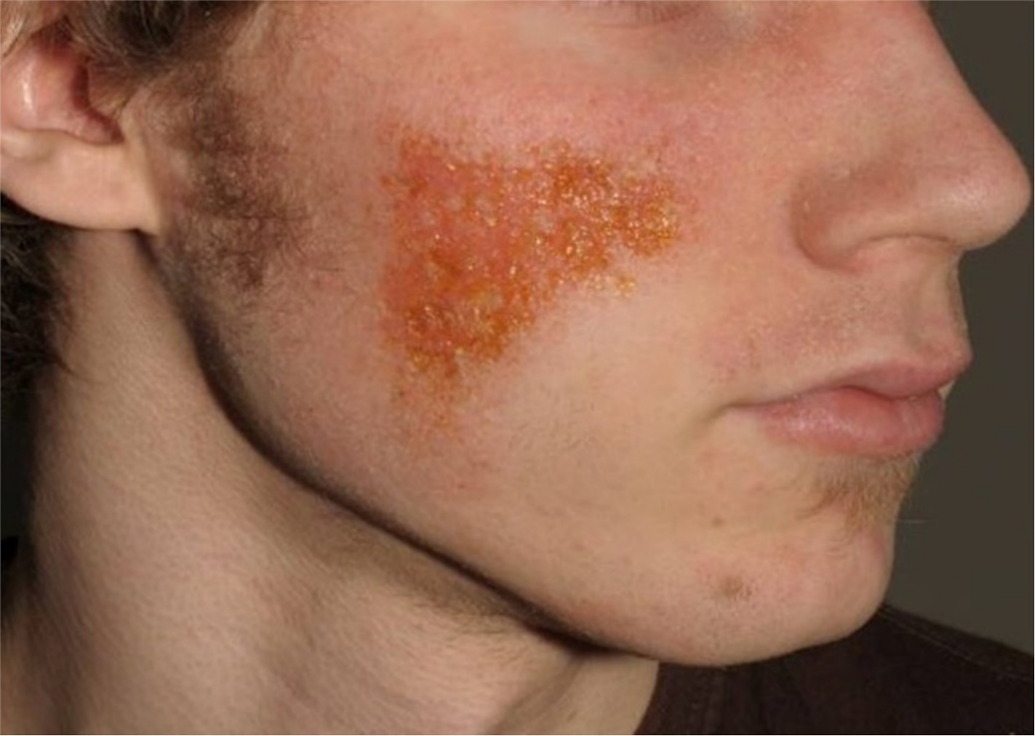
**Secondarly infected atopic dermatitis on the face of an adolescent.**

The disease development is believed to be strongly influenced by the host’s immune response. Immunological factors (e.g. Th_2_-shift) are thus understood to play major role in the pathogenesis of the disease. Changes in the balance of the microbiome and the host’s cutaneous immune response have been shown to aggravate atopic dermatitis and lead to secondary skin infections [17] (Figure 2). During flares, patients with atopic dermatitis show an altered skin microbiome. After adequate therapy the skin microbiome has been shown to regenerate. In atopic dermatitis the effect of narrowband UV-therapy has been documented quite well and shows an increase in microbial diversity [18, 19]. It has been postulated that reasons for deviations in the skin microbiome are based on endogenous (e.g. genetic polymorphisms in antimicrobial peptides of the native immune system) or exogenous (e.g. excessive hand washing and irritation) factors [20]. However it remains largely unclear whether the changes seen in the skin microbiome of are due shifts in the balance of the immune response or occur secondarily because of permeability barrier changes [21]. The role of the intestinal microbiota in the pathogenesis of atopic dermatitis has been investigated recently, but the establishment of the intestinal microbiome and its role for atopic dermatitis in early childhood remain uncertain [22].

### Decrease in the cutaneous microbial diversity

A hallmark of AD is the chronic, recurrent flaring of intensely itchy skin. In recent studies there has been a strong association between worsening disease severity and lower skin bacterial diversity in the absence of recent treatment. The microbiome shifts have been primarily localized to sites of disease predilection, e.g. folds or facial area. In contrast, intermittent or active treatment is associated with higher bacterial diversity.

**Figure 3:**
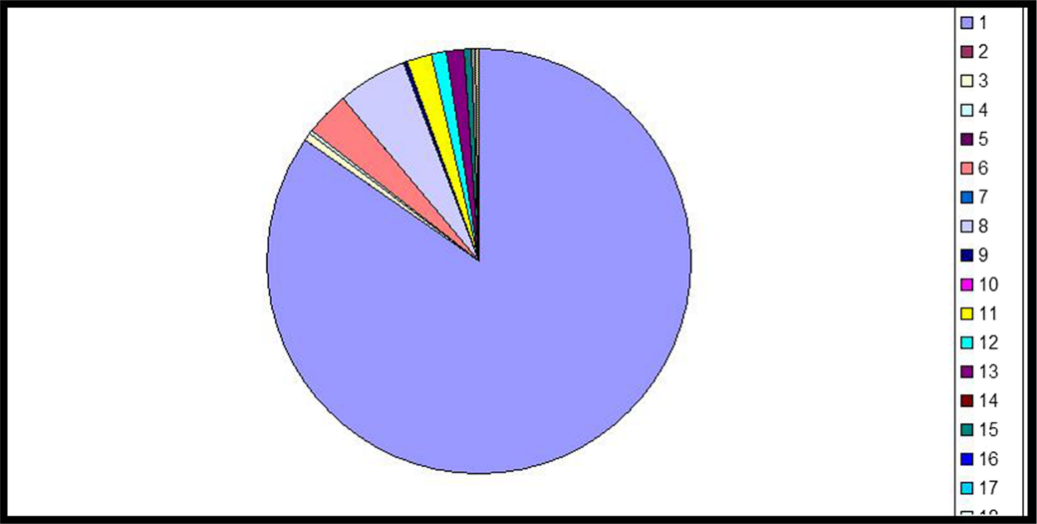
**Decreased cutaneous microbial diversity, colors indicate different bacterial phyla.**

**Figure 4:**
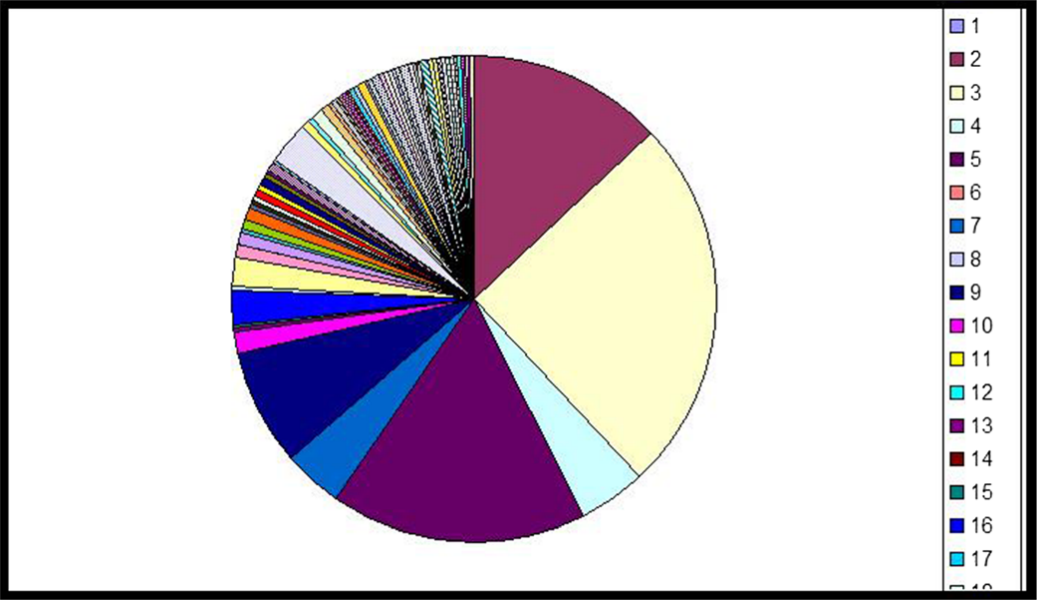
**Normal broad cutaneous microbial diversity.**

In one study intermittent-treatment flare patients who used sporadic treatments in the week preceding flare sampling, there was an early shift toward a diverse microbiome in the presence of active clinical disease suggesting that known effective treatments in atopic dermatitis (topical corticosteroids, topical calcineurin inhibitors and basic emollients) diversify the skin bacterial community [15] (Figures 3 and 4). It was shown however, that in the presence of active clinical disease during intermittent-treatment flares lesional skin requires continued intensive treatment to sufficiently reduce the inflammatory response. Increases in microbial diversity associated with treatments may be based on therapies that preferentially kill *Staphylococcus aureus* and consequently permitting the relative expansion of apathogenic bacteria. A possible other explanation is the promotion of microbes that control *S. aureus* predominance or a general reduction of skin microbes, which is followed by a prompt recolonisation with a community of broader diversity [21].

Many dermatologic disorders present with a typical clinical distribution and atopic dermatitis can be considered as a prime example. Correspondingly the site-specificity of skin bacterial communities proposes that not only do particular ecological niches of the skin favor the growth of certain bacteria, but that the local skin microbiome is important in the initiation and continuance of the disease.

### Role of Staphylococcus aureus

In recent years particular attention has been paid to the role of the Gram positive bacterium *Staphylococcus aureus* in atopic dermatitis [23]. There has been some evidence that during flares of atopic dermatitis the skin microbiome changes to more susceptible towards *Staphylococcus aureus* colonization [24]. In flares an increase of otherwise commensal Staphylococcus epidermidis colonisation and an appearance of Staphylococcus aureus colonization has been observed frequently [18]. It has been shown, that variations in the skin microbiome can modulate the interaction of genes and environment on the surface of the skin and thus have a proven impact on the skin's immune defense [25]. Recently, an example has been described how the host and cutaneous microbial flora join forces against pathogenic microbes. The usually apathogenic *Staphylococcus epidermidis* has been shown to inhibit pathogenic *Staphylococcus aureus* colonization and biofilm formation on the skin [26].

Current studies have shown that atopic dermatitis might at least partly have an underlying microbial etiology and clinical improvement was seen with antimicrobial treatments. At present the ruling consensus is that microorganisms have a secondary role, but only little is understood about their contribution and the pathogenesis. Many important questions are rising about the host-microorganism relationship and its relevance. Atopic dermatitis often occurs in characteristic areas of the skin such as the elbows and knee folds, which could be partly explained by differences in the skin microbiome. Whether these specificities are driven by the endogenous microbial community structure or is only an epiphenomenon due to secondary changes, e.g. changes in the skin barrier in atopic dermatitis (e.g. flaggrin mutations) or immunologic factors (e.g. Th2-shift), remains to be determined. However the characteristic changes in microbial colonization have awoken an interest in developing new diagnostic methods and treatments [27].

## Future perspectives

Microbes are traditionally considered as discrete causative agents, but this perspective is beginning to change. On human skin they exist within a rich milieu of other microbial species that can influence pathogenicity. Additionally to the known dysfunctions in barrier function of the skin and immunologic disturbances, evidence is rising that atopic dermatitis is also connected to a dysbiosis of the microbial community without an invading pathogen dominating the community.

Until now, the reported changes of cutaneous microbial diversity in atopic dermatitis have been quite characteristic for a particular disease state (stable or exacerbated) and possibly as a prognostic factor (disease development) and therefore may compose a potential diagnostic tool in the future. Novel molecular techniques to identify and quantify microbial organisms have to date proved to be sensitive and less-biased thus representing a possible subsidiary to clinical and microscopic diagnostics. Together with the clinical information these advances could point toward bacteria as new diagnostic and therapeutic targets in atopic dermatitis.

Molecular genetics have dramatically changed our vision of the micro-organisms colonizing the human skin. Important new questions have arisen in atopic dermatitis: the host micro-organisms interaction and its relationship to the pathogenesis of the disease, the concept of a dysbiosis and potential therapeutic strategies and the role of anti-microbial treatments. While it is clear that many of the dominant organisms (including Staphylococcus species) form a large part of the skin microbiome, very little is still understood of rare or transient organisms in atopic dermatitis. And, it is still unclear what factors are driving the changes in skin microbiome during flares of the disease [28]. Novel fields in skin microbiome research include the role of micro-organisms other than bacteria, e.g. fungi or viruses, in atopic dermatitis. For example there have been some reports of microbiome shifts in cutaneous commensal fungi (Malassezia species, 18S ribosomal RNA techniques) in atopic dermatitis [29].

However, there is emerging a new understanding of the skin microbiome in atopic dermatitis, which has proven to more complex and interesting than previously assumed. Future molecular genetic studies may solve several aspects of the pathogenesis of the disease. The investigation of the patient’s skin microbiome may also have a foothold in the clinician’s diagnostic repertoire and treatment of atopic dermatitis.

## Consent

Written informed consent was obtained from the patient for the publication of this report and any accompanying images.
